# PPoma Review: Epidemiology, Aetiopathogenesis, Prognosis and Treatment

**DOI:** 10.3390/diseases6010008

**Published:** 2018-01-11

**Authors:** Thais Ligiero Braga, Ralph Santos-Oliveira

**Affiliations:** Brazilian Nuclear Energy Commission, Nuclear Engineering Institute, Rio de Janeiro 21941906, Brazil; thaisblig@gmail.com

**Keywords:** cancer, oncology, imaging, radiopharmacy, nuclear medicine

## Abstract

Generally, pancreatic polypeptide-secreting tumor of the distal pancreas (PPoma) is classified as a rare tumor, and may occur sporadically or be associated in families or with multiple endocrine neoplasia type 1 (NEM 1). It grows slowly, reaching large dimensions at the time of diagnosis and the symptomatology is fundamentally due to the mass effect, causing either non-specific abdominal pain or symptoms suggestive of obstruction of the pancreatic or biliary duct. Therefore, when detected, they are usually malignant, with metastases mainly in the liver. The combination of serum analysis of increased levels of chromogranin A and pancreatic polypeptide and pancreastatin is very useful with a sensitivity of up to 95%. However, in addition, scintigraphicexams with somatostatin analogues should be performed to better clarify the diagnosis. Surgical resection is the treatment of choice, despite surgical difficulty and because they are generally palliative due to the metastases. Surgeries for tumor volume reduction are also performed to relieve symptoms. Chemotherapy commonly uses streptozotocin and somatostatin analogues to treat residual disease. Unfortunately, the survival rates are still very low, less than 10%, and if metastases already exist, this percentage drops to 3%.

## 1. Introduction

Historically, the first time that the pancreas was spoken of was by a Greek anatomist and surgeon known as Herophilus (335–280 BC). However, it is only much later that Ruphos, another Greek anatomist, gave the pancreas its name [1]. In antiquity and the middle ages, knowledge about the anatomy of the pancreas was very limited and its function was completely unknown. Significant progress was first made in the seventeenth and eighteenth centuries. Johann Georg Wirsung, the prosector of the University of Padua, discovered the main pancreatic duct, and Giovanni Santorini discovered the accessory duct. Regnierde Graaf was the first to perform a pancreatic exocrine study, and Paul Langerhans’s discovery of pancreatic islets was in 1869. After the discovery of Langerhans, the first steps toward recognizing the pancreas as an endocrine gland were done. The twentieth century brought the discovery of insulin and other pancreatic hormones. Early, pancreatic studies led to crucial advances in scientific knowledge and were recognized, among other things, with seven Nobel Prizes. The first of these went to Ivan Pavlov in 1904 for his work on the physiology of digestion. The most recent was awarded to Günter Blobel in 1999 for discovering signaling mechanisms that govern the transport and localization of proteins within pancreatic acinar cells [2,3,4].

The pancreas consists of a gland that has an elongated, pointed shape measuring about 15 cm and which is located below the stomach, between the duodenum and the spleen. It is part of the digestive system and the endocrine system of vertebrates, and consists of endocrine cells introduced into exocrine tissue [5].

The endocrine part of the pancreas is made up of clusters of cells known as the islets of Langerhans. In these islets, there are four types of cells (alpha, beta, delta and PP) that although difficult to differentiate, can nevertheless be classified according to the material that they secrete. PP cells, which account for less than 2% of the islets, produce the pancreatic polypeptide (PP) whose function is to inhibit the exocrine pancreas and reduce the release of somatostatin. In addition to enzymes and hormones, the pancreas also secretes large amounts of sodium bicarbonate, which is intended to protect the duodenum from acid from the stomach by neutralization [5,6]. Although the pancreatic polypeptide (PP) is secreted by the PP cells in the endocrine pancreas, these cells are also spread throughout the exocrine pancreas. It consists of 36 amino acids and has a molecular weight of approximately 4200 Da, being a cholecystokinin antagonist, suppressing pancreatic secretion and stimulating gastric secretion [7]. Its secretion in humans is increased after a protein meal, fasting, exercise and acute hypoglycaemia, and is reduced by somatostatin and intravenous glucose. The role of PP is unknown, but effects on hepatic glycogen levels and gastrointestinal secretions, such as the stimulation of gastric and intestinal enzyme secretion and inhibition of intestinal motility, have been suggested [8,9] The highest concentration of PP cells is in the head of the pancreas, where 90% of all PP cells are located [6]. PP was first reported in 1972 when it was simultaneously isolated by two independent laboratories [10,11].

Pancreatic neoplasms are divided into two groups: exocrine pancreatic cancer (approximately 94%), represented mainly by adenocarcinoma, and endocrine pancreatic cancer (approximately 6%), represented by carcinoid tumors (well-differentiated neuroendocrine tumor) and neuroendocrine tumors (NETs) [12,13,14]. Neuroendocrine tumors (NETs) are also found in the stomach and intestine, but are heterogeneous in their morphology, function and biology. Therefore, the WHO classification distinguishes gastroenteropancreatic NET according to its location, histopathology, proliferative activity, extension, functional activity and hereditary antecedents [15]. Most cases of pancreatic neuroendocrine tumors affect the right side of the organ (the head); gastrinomas, pancreatic polypeptide (Secretory Tumor of the Distal Pancreas) and somatostatinomas are more frequent, whereas insulinomas and glucagonomas predominate in the centre (body) and in the left side (tail) [16]. Its location in the retroperitoneum is associated with its natural history, and forms a great challenge for early diagnosis. All pancreatic neuroendocrine tumors are considered malignant and have the potential of metastasis; for this reason, its curative treatment is still restricted to a minority of patients; in addition, lesions are generally unresectable and 50% are metastatic at diagnosis; in the case of PPoma, the number of metastatic tumors at diagnosis rises to 90% [12,13,17].

Clinically, neuroendocrine tumors (NET), including endocrine pancreatic tumors, can be divided into functional and non-functional tumors [12]. Non-functional tumors account for 50–60% of all NETs and include those which are clinically quiet but secrete a predominant substance, e.g., the PPoma, which make up about 50% of all pancreatic NETs (PNET). Tumors are non-functioning or are diagnosed incidentally (for example, by endoscopy) or due to the nonspecific symptoms of mass effects, such as enlarged liver, obstruction of the pancreatic duct, or jaundice being diagnosed mainly in the head of the pancreas and between the 5th and 6th decade of life. PPoma presents a shorter overall survival time than functioning tumors [13,18,19]. Also, differentiation between a non-functional pancreatic NET and an adenocarcinoma of the pancreas is extremely important because the prognosis is clearly different; usually, NETs have a better prognosis than exocrine tumors of the pancreas. However, because the differences between these two types of tumors may be subtle, it is often difficult to distinguish between them [19,20,21,22,23].

## 2. Epidemiology

Cancer is considered a worldwide public health issue, especially among developing countries, according to the World Cancer Report database of the International Agency for Research on Cancer (IARC) of the World Health Organisation (WHO). It is estimated that in the coming decades, the impact of cancer on the population will correspond to 80% of the more than 20 million new cases estimated for 2025 [24].

In this scenario, we have the pancreatic cancer. This type of cancer is rare before the age of 30, but becomes more common from the age of 60. According to the International Union Against Cancer (UICC), cases of pancreatic cancer increase with age: from 10/100,000 inhabitants between 40 and 50 years to 116/100,000 inhabitants between 80 and 85 years. The incidence in the Brazilian population is more significant in men, with 8710 deaths having occurred in 2013, of which 4373 were men and 4335 were women. Between 62% and 82% of the patients present with tumors originating in the gastrointestinal tract [12,25]. When evaluating endocrine tumors of the pancreas (PNET), the annual incidence is even lower, approximately one in 100,000 or 1–2% of all pancreatic neoplasms [26,27].

Nonfunctional pancreatic tumors like Ppoma, are the most common PNETs, representing approximately 50–60% of all PNET. These tumors are diagnosed more frequently between the 5th and 6th decade of life. They occur more frequently in the head of the pancreas and most of these tumors are malignant. Non-functional PETs are tumors that do not present the clinical syndrome of hormonal hypersecretion, which means that most of them are silent. These tumors may occur in 20–40% of patients with MEN (multiple endocrine neoplasia) and they can also be found in Von Hippel-Lindau’s disease. In fact, PNETs whose are associated with this disease are almost always non-functional tumors [28,29].

**Figure 1:**
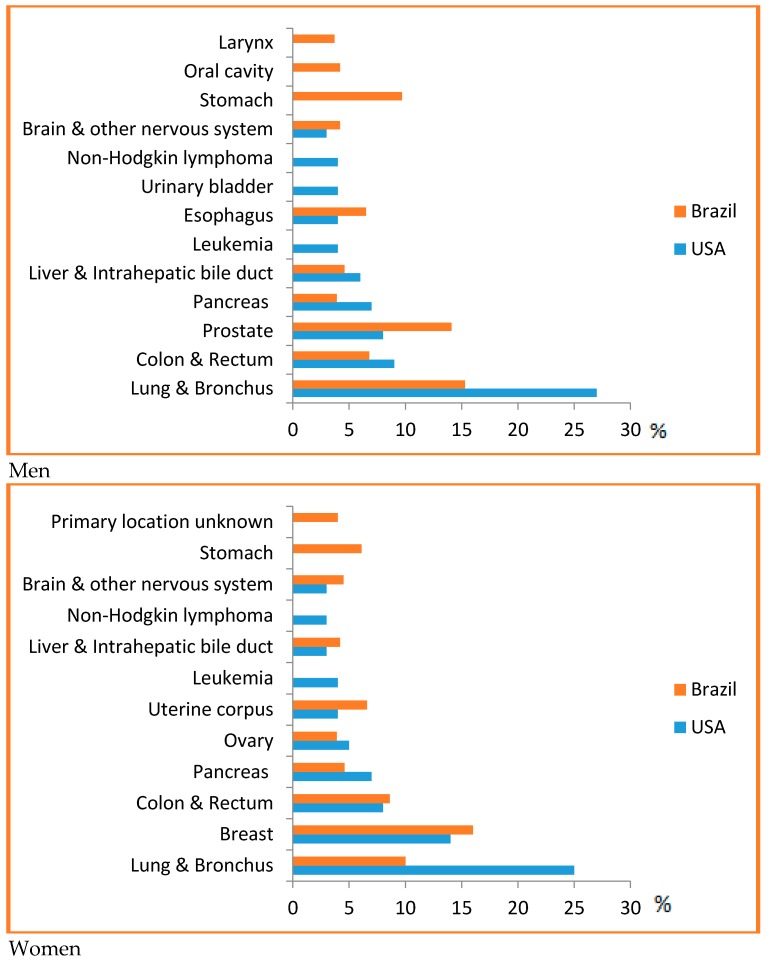
Top causes of cancer death, by sex in Brazil, 2008 [31] and the US [14]—Estimates for 2017. As estimates are rounded to more than ten cases, the exclusive cases of basal cells and squamous cells are skin cancers and carcinoma in situ, except for bladder urinary tract infection. Brazilian source: MS/SVS/DASIS/CGIAE/ Sistema de InformaçãosobreMortalidade—SIM; MS/INCA/Conprev/Divisão de Informação e análise de Situação. American Source: AMERICAN CANCER SOCIETY. Cancer Facts & Figures 2017. Atlanta; 2017 [14].

PPoma is considered a rare disease, since it represents less than 1% of non-functional neuroendocrine tumors [30]. For all combined stages, the relative five-year survival rate is 8%. For the small percentage of people diagnosed with local disease (9%), the five-year survival is only 29%. About half (52%) of patients are diagnosed at an advanced stage, for which the five-year survival is 3% [14]. Most of this situation is due to the fact that it is difficult to detect, and pancreatic cancer has a high mortality rate because of the late diagnosis and aggressive behavior. In Brazil, it accounts for about 2% of all cancers diagnosed and 4% of all cancer deaths [25]. It is considered one of the main causes of cancer death in Brazil, being the ninth highest cause among men and the sixth highest cause among women [31]. In the US, it is estimated that 53,670 new cases of pancreatic cancer will be diagnosed in 2017 and it is considered the third leading cause of cancer death in men and women in US, since they are associated with a wide range of other tumors, for example, ovarian, breast, endometrial, bladder, prostate, or esophageal cancer, with a predicted 43,090 deaths in 2017, and a risk of one in 67 (Figure 1) [14,32,33,34].

From 2004 to 2013, pancreatic cancer incidence rates increased by about 1% per year in Caucasian individuals, but were stable in afro descendant [14]. Regarding mortality rates, an increase of 0.3% per year was observed in Caucasian men from 2005 to 2014, and a decrease of 0.5% per year was observed in afro descendants both men and women in the same period [14]. The relative five-year survival rate has increased from 3% in the mid-1970s to 9% in the most recent period (2006–2012) [35].

## 3. Risk Factors

Some risk factors for pancreatic cancer have been identified and an important association has been found in relation to age, since incidence rates increase progressively with age, with more than 80% of cases of pancreatic cancer occurring in individuals aged between 60 and 80 years [36].

**Figure 2:**
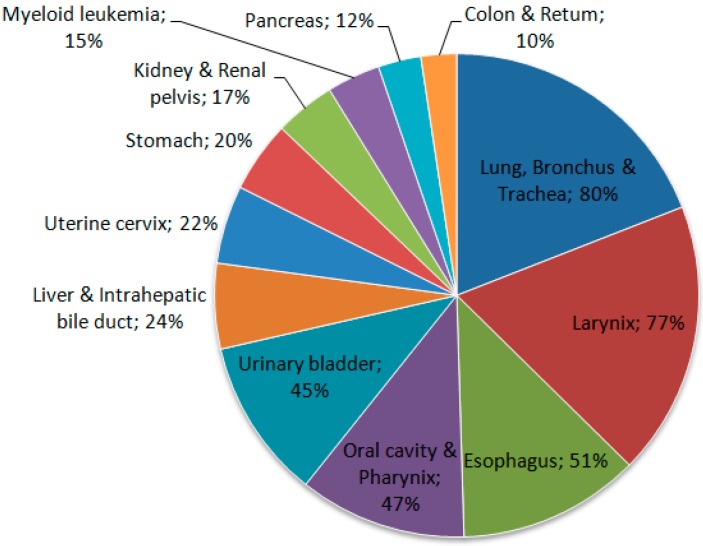
Proportion of cancer deaths attributable to smoking in adults aged 35 years and over, USA, 2011. Source: Adapted from Siegel et al. 2015 [28].

Tobacco use is one of the main risk factors for pancreatic cancer, showing that it clearly plays an important role in this neoplasia [31]. Some cigarette smokers have a risk of developing pancreatic cancer that is about twice as high as that for individuals who have never smoked. The use of smokeless tobacco also increases the risk (Figure 2) [33].

Other risk factors include a family history of pancreatic cancer, a personal history of chronic pancreatitis or diabetes mellitus, obesity, a diet rich in meat and fats, and low levels of serum folate. Excessive alcohol consumption can increase the risk. Individuals with Lynch syndrome and certain other genetic syndromes, as well as *BRCA1* and *BRCA2* mutation carriers, are also at an increased risk [14,37]. In addition, approximately 7–10% of patients show a familial predisposition for pancreatic cancer [38]. Consuming fruits and vegetables reduces the risk of pancreatic cancer [39].

Regarding specific risk factors to PPoma, there are some that deserve attention: (i) high intake of saturated fat, (ii) malignancy and associated impairment in glucose metabolism, (iii) thyroid dysfunction, (iv) alcohol consumption, (v) cigarette smoking, (vi) chronic medical conditions, like diabetes mellitus, and (vii) environmental factors, like radiation [40].

## 4. Aetiopathogenesis

According to the distribution the cells in the islets of Langerhans of the pancreas, there is a predominance of certain tumors. The head is the area of the gland with the greatest density of PP cells, which produce PP, and give rise to PPoma, The current targets of screening include identification of early pancreatic cancer, as well as the two most important precursor lesions intraductal papillary mucinous neoplasm and high grade pancreatic intraepithelial neoplasia. In other words, it is more likely that endocrine pancreatic tumors originate from the already differentiated cells of the islets of Langerhans [6,16,18,23,26,27].

Pancreatic neuroendocrine tumors (PNET) are uncommon neoplasms, with an annual incidence of approximately one in 100,000; these are distinguished by several characteristics and account for only 1–2% of all pancreatic malignancies [26,41]. Neuroendocrine cells (derived from Enterochromaffin cells or Kulchitsky cells) are distributed throughout the body. They exhibit similar biochemical and functional behavior, with the possibility of taking up precursors of amines and their decarboxylation; therefore, they also receive the designation of cells of the amine precursor uptake and decarboxylation (APUD) system. The embryological origin appears to be the gastroenteropancreatic tract, not the neural crest as previously thought. Neoplasms from cells of the APUD system have the ability to synthesize and secrete polypeptides that have specific endocrine hormone activity, such as the PPoma [12,18,42,43].

Clinical behavior and the occurrence of well-defined syndromes depend on the production of substances secreted by tumor cells (insulin, glucagon, etc.). As PPoma is a non-functioning (non-secreting) tumor, it has no clinical manifestation until it is large enough to cause compressive symptoms, despite having high serum levels of pancreatic polypeptides. However, histologically, non-secretory tumors are indistinguishable from secretors, but they do not have the characteristic of the production of molecules with hormonal activity [18,44,45].

The 2010 World Health Organisation (WHO) classification of neuroendocrine neoplasms arising in the digestive system (gastroenteropancreatic NETs) separates these tumors into two broad categories [46]. The first includes well-differentiated neuroendocrine tumors, which show a solid, trabecular, gyriform, or glandular pattern, with fairly uniform nuclei, salt-and-pepper chromatin, and finely granular cytoplasm. These tumors have been traditionally referred to as carcinoid tumors when arising in the tubular gastrointestinal tract or pancreatic neuroendocrine (islet cell) tumors, where the PPoma fits and has a high degree of malignancy [38,47]. The second includes poorly differentiated neuroendocrine carcinomas, which are high-grade carcinomas that resemble small cell or large cell neuroendocrine carcinoma of the lung [48]. Poorly differentiated neuroendocrine carcinomas are often associated with a rapid clinical course; as such, their clinical behavior is similar to that of small cell carcinoma of the lung, and they are treated similarly, using platinum-based chemotherapy [49,50].

In contrast, well-differentiated neuroendocrine tumors of the digestive system generally have a much better prognosis. However, these tumors are not a homogeneous group, but instead display a spectrum of aggressiveness. Even in the presence of liver metastases, some patients may survive for many years. Within the subgroup of well-differentiated NETs, morphology alone cannot predict tumor behavior. Proliferative rate, as assessed by mitotic count and/or Ki67 labelling index, is of prognostic significance, independent of tumor stage [49,50]. The WHO classification separates well-differentiated gastroenteropancreatic NETs into low-grade (G1) and intermediate grade (G2) categories based upon proliferative rate (Table 1). All poorly differentiated neuroendocrine tumors are high-grade (G3) neuroendocrine carcinomas according to this classification scheme. However, there is a small subset of tumors that displays a high proliferation rate but well-differentiated morphology [46].

The term “Neuroendocrine tumor grade 3 (G3)” has been used for this category but is not advised, since neuroendocrine tumors are, by definition, well-differentiated. Reproduced with permission from: WHO Classification of Tumors of the Digestive System, 4th edition, Rindi GAR et al. [46]

## 5. Clinicopathological Presentation

Most of the signs and symptoms of neuroendocrine pancreatic tumors are caused by the excess hormones that tumors release into the bloodstream. In PPoma, there is an overproduction of the pancreatic polypeptide, which helps to regulate both the exocrine and endocrine pancreas. However, this excess hormone does not usually cause any identifiable hormonal syndrome, since the hormone is biologically inactive. Thus, they grow slowly, reaching large dimensions at the time of diagnosis; the symptomatology is mainly due to mass effect, causing abdominal pain, jaundice, altered general condition, palpable mass, diabetes mellitus and weight loss, with some patients also presenting gastrointestinal bleeding and watery diarrhea. The latter has been associated with very high levels of PP; in addition, most cases of PPoma are malignant when diagnosed (90%). Jaundice (yellowing of the skin and eyes) can sometimes facilitate early diagnosis. A recent report suggests the association of PPomas with diabetes mellitus, demonstrated in five case reports where patients had an improvement or resolution of diabetes after resection of the tumor [13,17,18,33,51,52]. The literature also reports on the use of genetic algorithms in the development of genetic resources in the United States. The evolution of patients with pancreatic neuroendocrine tumors is quite variable. Indolent tumors can lead to the patient being asymptomatic, even without specific treatment. Other patients require therapy due to compressive symptoms, or hormone peptide production. The predominant site of metastasis is the liver [3,17,18]. In PPoma, enlargement of the liver is one of the main symptoms used for its detection [33].

Multiple endocrine neoplasia type 1 (MEN-1) was first described in 1954 by Wermer [53]; for this reason, it is also known as Wermer syndrome [54,55,56]. MEN-1 syndrome is rare, occurring with a prevalence of 0.001–0.25%, and reaches all age groups, presenting an identical distribution in both sexes. It results from inactivation of the tumor suppressor gene MEN-1, has autosomal dominant transmission and the penetrance is almost 100% with age. It is observed that penetrance increases with age, because it is estimated that penetrance is 3% at 20 years of age in non-functional pancreatic neuroendocrine tumors, increasing to 53% at 80 years. The term multiple endocrine neoplasia (MEN) encompasses distinct, genetically determined disorders associated with the process of hyperplasia or neoplasia in two or more endocrine glands of the same patient. The major MEN types are multiple endocrine neoplasia type 1 (MEN-1), primarily affecting the parathyroid, pancreas and pituitary gland, and multiple neoplasia type 2 (MEN-2), which involves the thyroid (medullary carcinoma), parathyroid and adrenal medulla (phaeochromocytoma). However, von Hippel Lindau’s syndrome, neurofibromatosis type 1 and the Carney complex are still part of the MEN spectrum [12,54,57,58,59].

The gene related to MEN-1 was identified in 1997 and is located on the long arm of chromosome 11 (11q13). It consists of ten exons, spans approximately 9 Kb and encodes a nuclear protein of 610 amino acids, called MENIN or girl. Since the characterization of the *MEN-1* gene, different studies have demonstrated multiple mutations causing familial and sporadic MEN-1. Approximately 261 germline mutations were reported in independent families [12,54]. Mutation of the *MEN-1* gene is the most common genetic alteration found in pancreatic neuroendocrine carcinomas (PNC), but with distinctly different frequencies between them; PPoma is present in 18–44% of the cases [60]. In the case of insulinomas (7%), the first genetic key [61] has been revealed. The fact that mutations in *MEN-1* are found in non-functioning PNC is not surprising when considering that non-functioning PNC are common in patients with multiple endocrine neoplasia type 1 (MEN-1) and higher morbidity and mortality are associated with MEN-1, not to mention that the average life expectancy for patients with these tumors was lower than that of patients with MEN-1 who did not present with PNC tumors. In the last few decades, MEN has aroused special attention, since the inheritance characteristic of these syndromes offers a unique opportunity to the study of genes involved in the process of carcinogenesis [12,62].

Rarely, neuroendocrine tumors are associated with von Hippel Lindau disease, characterized by pancreatic islet tumor, where the PPoma is located. This syndrome is characterized by inactivating mutations of the *VHL* gene (3p25) encoding the elonginprotein [12,54].

## 6. Diagnosis

The vast majority of non-functional pancreatic neuroendocrine tumors (PNETs) are diagnosed as a result of nonspecific abdominal pain or symptoms of pancreatic or biliary duct obstruction. Because of this, non-functional PNETs tend to be larger when detected (5.9 cm), have a higher rate of metastasis (60% in general, but rising to 90% in the case of PPoma) and a worse prognosis (33% survival in 5 years) [52].

**Figure 3:**
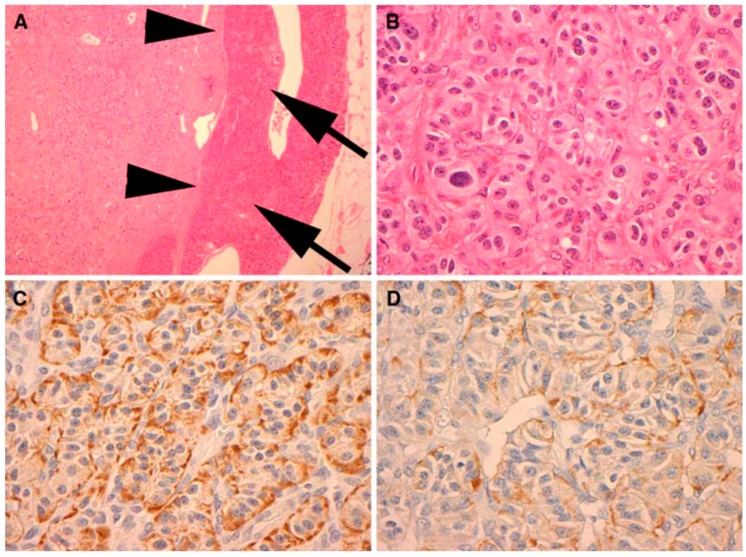
Immunohistochemical (IHC) analysis to PP (Novocastra NCL-PPp, dilution 1/500) (**A**). All PPomas were not encapsulated but well circumscribed and separated from the adjacent normal pancreas (arrows) by a smooth pseudocapsule (arrow heads) (**B**). All PPomas exhibit characteristic features typical of neuroendocrine differentiation, including speckled chromatin (“salt and pepper”) and a nested and trabecular architecture. Original H & E 600× amplification (**C**). All PPomas show strong diffuse positive staining for general markers of neuroendocrine differentiation including chromatin immunohistochemistry. IHC original amplification 600× (**D**) [30]. All PPomas showed positive labelling for pancreatic peptide by immunohistochemistry. Immunohistochemistry of pancreatic polypeptide, original amplification 600× [30] (Figure 4).

**Figure 4:**
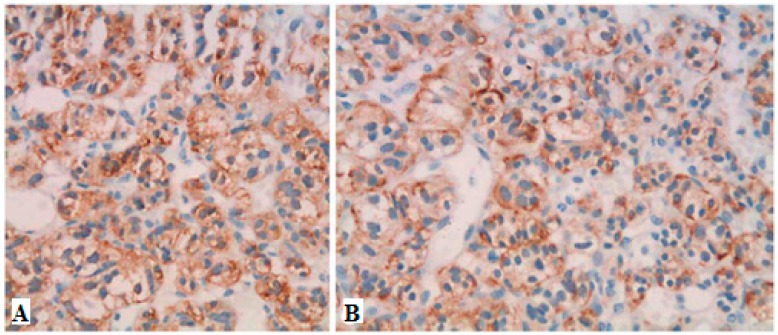
Immunohistochemistry showing strong positive staining for chromogranin A (**A**) and PP (**B**) in a case of PPOMA [26].

Neuroendocrine tumors comprise a large family of neoplasms of neuroectodermal origin or pluripotent cells. They are characterized by the presence of neurosecretory granules identified by electron microscopy or by immunohistochemical study of chromogranin, synaptophysin, specific neural enolase or PGP 95 (Protein of the product gene 9.5—Protein Gene Product 9.5). Biomedical evidence shows that abnormal serum concentrations of nonspecific neuroendocrine markers, such as chromogranin A (60–100%), pancreatic polypeptide (PP) (25–70%) and pancreastatin, are the main findings. Chromogranin A, called secretagranin I, is from a group of proteins present in several neuroendocrine tissues, and is a good marker for both neuroendocrine tumors and pancreatic islet cell carcinoma, as well as multiple endocrine neoplasia. The reference range in the serum is from 10 ng/mL to 50 ng/mL. The dosage of chromogranin-A should be assessed annually for follow-up, but this marker is found to be 60–80% higher in patients with neuroendocrine tumors, regardless of the primary site. PP dosage is determined by the radioimmunoassay technique, using plasma EDTA, with a standard value lower than 300 pg/mL. For the most part, the co-association of elevated PP levels in PNETs and other hormones has maintained its value in the diagnosis and follow-up of patients with functional and non-functional PNETs. Therefore, PP is a good marker to test in all cases of suspected PNET, in addition to the hormones suggested by a clinical syndrome, if present. However, this parameter has some interferences like other PNETs, nesidioblastosis, PP cell hyperplasia and renal dysfunction. Pancreastatin, a pancreatic peptide derived from chromogranin A, has a counter-regulatory effect on insulin action, and is also useful for monitoring the effects of therapy and the progression of PPoma [63]. In this paper, we present the results of a study of the results obtained in the literature based on literature review of Ppoma [12,17,18,44,63,64,65,66]. Figure 3 and Figure 4 show some immunohistochemical evidence of PPoma cases.

The use of radiopharmaceuticals to achieve nuclear medicine imaging is the best way to anatomically and functionally identify an organ. This technique allows the characterization of functional and metabolic parameters in vivo and in a non-invasive way, by the administration of radiopharmaceuticals ready for use or coupled to other molecules. The information provided may assist in clinical reasoning in several situations in which anatomical imaging methods are limited (e.g., small lymph node infiltration or residual tumor screening after treatment). Thus, the diagnostic applications of nuclear medicine in oncology include the detection and characterization of the primary lesion, staging and control of the therapeutic response [12,67,68].

Radiopharmaceuticals are generally administered via IV (intravenous), awaiting concentration on the target tissue prior to acquisition of the images. The images obtained in the scintillation chamber reflect the distribution of the radiopharmaceutical in the patient and can be flat or tomographic single photon emission tomography (SPECT) [12,69,70].

In the 1960s, in a study of hypothalamic factors, somatostatin [71] was discovered and later identified in the delta cells of the islets of Langerhans and similar cells of the gastrointestinal tract and central nervous system. This hormone is a cyclic peptide containing 14 amino acids (aa) and belongs to a group of peptides including the original somatostatin (S-14), an enlarged molecule of 28 aa (S-28), and an initial 12 aa fragment of somatostatin 28 [S-28 (1–12)] [20]. These bind cellular receptors termed somatostatin receptor (SSTRs) in normal and tumor cells. Five SSTR subtypes were isolated, cloned and sequenced [72], and subtype 2 was found in PPoma [17]. Somatostatin inhibits the release of various hormones such as those from the pancreas [73]. Therapeutic uses of this polypeptide include blocking hormone release in secretory endocrine pancreatic, carcinoid and GH-secreting adenomas [74]. Therefore, knowing these properties, somatostatin analogues (octreotide and lanreotide) are labelled with radioactive substances for the acquisition of SPECT images.

**Figure 5:**
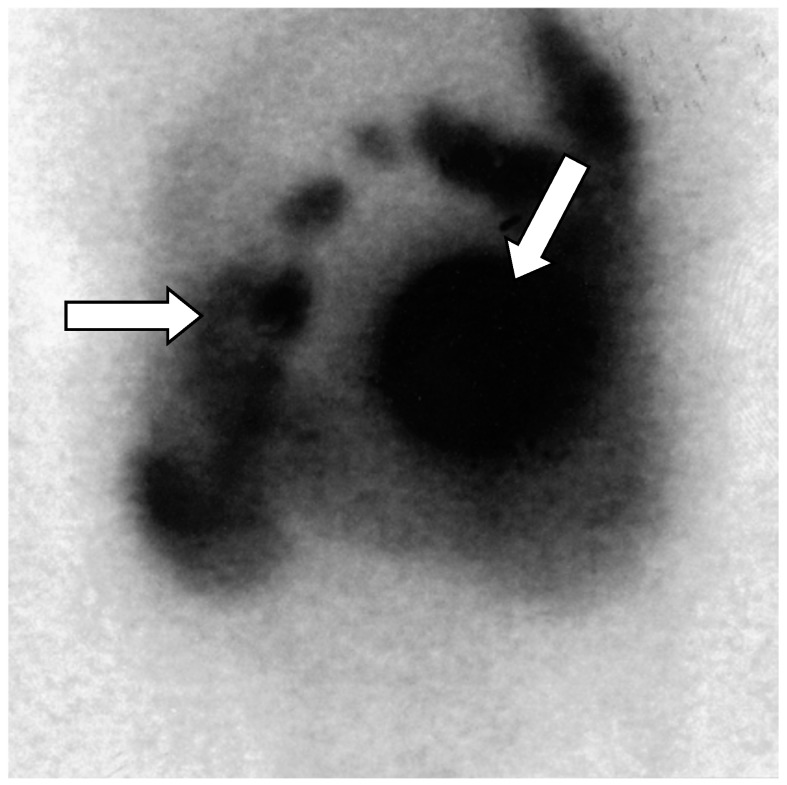
Octreoscan scintigraphy (Octreotide labelled 111-Indium _^111^ In-DTPA-octreotide) showing tumor-free uptake of the pancreatic head (arrows). There is normal absorption of the marker by the liver, spleen, and kidney. Normal excretion of non-intestinal radioactivity is observed [18].

Octreotide conjugated to indium-111-labeled diethylene triaminepentaacetic acid (DTPA) (Octreoscan: 111 In-DTPA-octreotide) has a high affinity for somatostatin receptors (mainly subtypes 2 and 5) expressed in several neuroectodermal lineage tumors. This enables positive images to be acquired for PPoma (Figure 5), showing progression of the tumor or new lesions, but other options should be considered for PPoma tumors. The sensitivity of the test is dependent on the expression of somatostatin receptors [12,17,75].

Scintigraphic exams with somatostatin analogues of the pancreas can be performed every three years. These tests are not performed annually because it is known that, mainly in the case of pancreatic tumors, its diagnosis is based almost exclusively on biochemical screening by the detection of Chromogranin-A, since the abnormality of pancreatic hormones precedes the radiological detection of these tumors by at least five years [54].

## 7. Prognosis

PPoma remains a reserved prognosis neoplasm due to its aggressive biological power, both locally and in relation to the spread of the disease [36]. The poor prognosis of this neoplasm is, in part, related to the difficulty of early detection. When the first symptoms appear, the majority of the patients present at an advanced stage of disease, usually metastatic, where curative surgery is no longer possible [13,76]. In this case, we pay attention to some initial symptoms, such as epigastric malaise, diarrhea or jaundice is crucial and should not be neglected [12,13,17,33,76].

Surgical resection is only performed in approximately 20% of cases, although it is the treatment of choice. Surgeries for tumor volume reduction are also performed to alleviate the effects of mass and malignant behavior. Complementary therapies are generally ineffective. Unfortunately, there is currently no sensitive and effective screening test for the early detection of PPoma or even to identify individuals predisposed to the development of this neoplasia [14,18,36].

## 8. Staging

**Figure 6:**
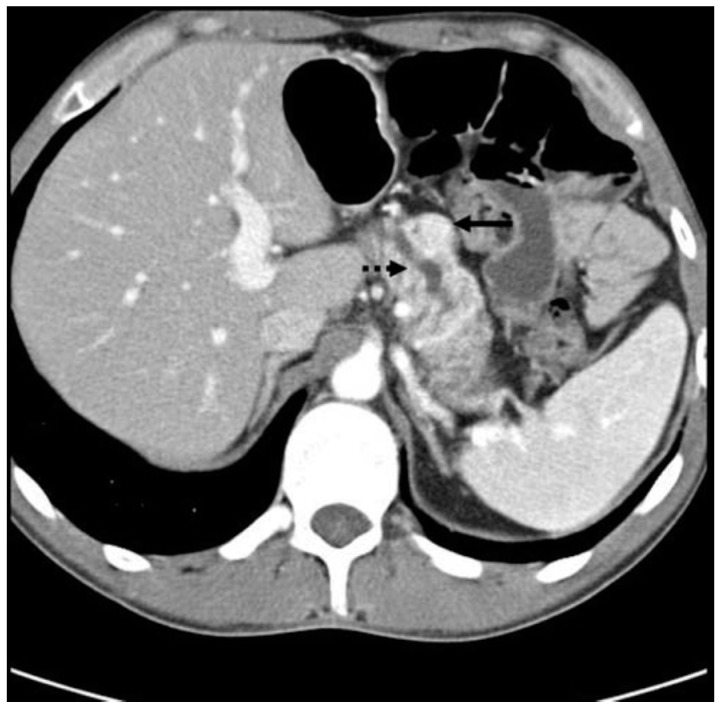
CT scan showing a PPoma of the head (solid arrow) of the pancreas causing pancreatic duct dilation (dashed arrow). The tumor is a discrete lesion, with well-defined borders, and shows contrast enhancement in the arterial phase [30].

**Figure 7:**
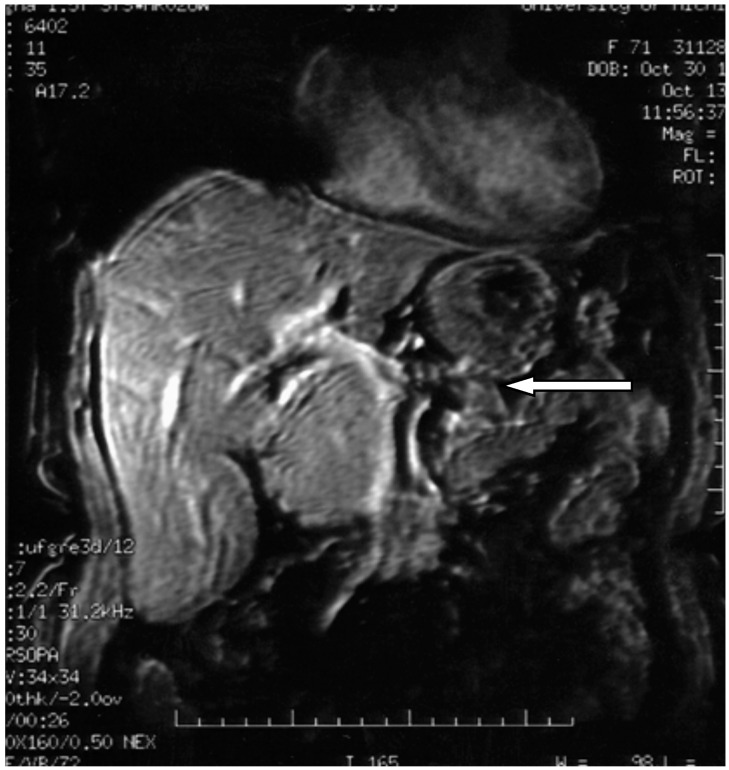
3D axial magnetic resonance imaging showing pancreatic polypeptide-producing tumors in the head of the pancreas (arrow) [18].

**Figure 8:**
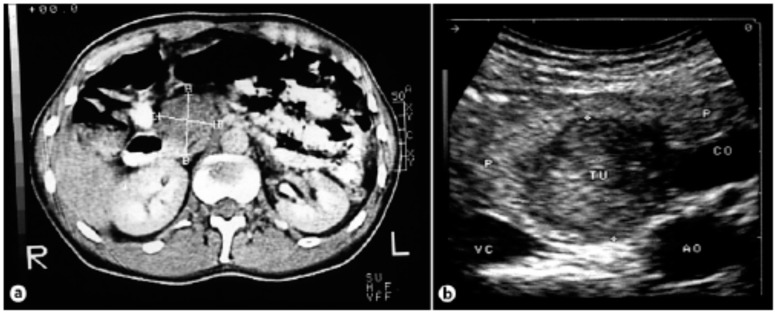
(**a**) Computed tomography of the abdomen showing a tumor of the pancreas 4.2 × 3.3 cm in size. (**b**) Intraoperative ultrasound showing a hypoechogenic tumor [26].

Staging must be performed from two anatomical and functional points of view, besides TNM. Anatomic staging should be performed by computed tomography (CT) of the thorax, abdomen and pelvis, and nuclear magnetic resonance imaging (MRI) of the liver may be useful in the characterization of liver metastases (Figure 6, Figure 7 and Figure 8). Upper digestive endoscopy with ultrasonography (US) helps to detect small lesions, allowing needle biopsy. Octreoscan, restricted to some centers, is an important staging test, and is important for in vivo evaluation of tumor expression of somatostatin type 2 receptors. In-111-labeled octreotide scintigraphy in combination with conventional imaging (CT or MRI), has improved the detection of primary and metastatic lesions, as well as selecting patients for radioactive therapies. However, research aimed at early detection should be an incentive for PPoma because of its high degree of malignancy. In patients with suspected MEN-1, US is used for evaluation of the parathyroid, since 90–97% of patients with this syndrome have primary hyperparathyroidism. Bone scintigraphy should be performed in patients with suspected bone metastases [17,68,77,78].

Functional staging involves plasma chromogranin A, which is increased in about 80% of cases, regardless of whether the tumor is functioning or not. This marker correlates well with the hormonal secretion of the tumor, and may not be related to the tumor mass. Its elevation may anticipate tumor growth detected by imaging tests. In addition, the pancreatic polypeptide-specific polypeptide should be dosed. Calcium and serum phosphorus, parathyroid hormone (PTH) and urinary calcium for characterization of hyperparathyroidism (present in MEN-1 in more than 90% of cases) should be performed at least once at diagnosis [17,52,78].

The first results published by the National Cancer Institute’s SEER (Surveillance, Epidemiology and End Results) program separated tumors by localized extension, regional extension or with distant metastases; these parameters were fundamental to initiate the first classification systems of neuroendocrine tumors NET) [79]. In 2006, a work group of the European Society of Neuroendocrine Tumors (ENETS_European Neuroendocrine Tumor Society) [80] developed and published a proposal for TNM classification of neuroendocrine tumors (NET) of the anterior intestine (stomach, duodenum and pancreas). This was the first TNM (Tumor, Nodes, Metastasis) classification to be developed for NETs that takes into account the distinctive growth patterns of these tumors and differentiates these tumors from other gastroenteropancreatic carcinomas (GEP). These classification systems complemented the WHO classification of GEP-NETs, using some aspects that had already been recognized as prognostically relevant. In the years following the publication of these proposed TNM classifications, the GEP-NET classifications of the previous gut and particularly pancreatic NETs were validated by several studies, and their biological relevance and the power to discriminate between the prognostic groups was broadly confirmed [81].

Later in 2009, the seventh edition of the TNM staging manual was published by the American Joint Committee on Cancer (AJCC) and the International Union Against Cancer (UICC) for all tumors, also including NET of all tumor sites, such as TNM of gastrointestinal carcinoids and pancreatic neuroendocrine tumors, which had not previously been included in the AJCC/UICC staging classifications. However, the seventh edition of the AJCC/UICC TNM classification does not apply to high-grade neuroendocrine carcinomas (large and small cells) and does not exactly follow the ENETS classifications for some anatomical sites, especially for tumors of the pancreas, stomach and appendix, although both TNM classifications overlap in the great majority [82]. No data are presented to justify the use of different staging parameters. The result is that there are now two parallel systems, each of which uses identical TNM terminology, but may refer to different types and extensions of disease for particular NETs. This discrepancy leads to much confusion among clinicians and probably limits the ability to compare research studies that use TNM staging as a prognostic factor or to stratify treatment [81].

Specifically, the AJCC/UICC and ENETS scores differ in the definitions of T stages for pancreatic tumors (Table 2). The ENETS system is widely used in Europe; in the United States of America, many professionals are already required to use the AJCC/UICC system. The confusion that will arise from these parallel systems is a problem. When a TNM classification is being applied in practice, it is important, therefore, to make clear which classification is being used. Another point is that, given the differences in these two systems, it is critical to document the underlying characteristics that contribute to the T-stage (tumor size, extent of invasion, etc.) classification in the pathology reports to allow translation between the ENETS and AJCC/UICC classifications [81]. Therefore, it is recommended that the pathologist’s report show, in addition to the TNM classification system, its basis for classification, which essentially includes the size of the tumor and the level of invasion [83].

## 9. Treatment

Surgery and chemotherapy are treatment options that aim to prolong survival and/or alleviate symptoms, but rarely produce a cure [14]. Thus, to define the treatment strategy for neuroendocrine tumors, the characteristics of tumor resectability are characterized, regardless of the condition of asymptomatic or symptomatic patients. PPomais are usually included in the classification of metastatic tumors and resectability, depending on the size of the tumor to be classified as resectable or unresectable. Resectable metastatic tumor resection of hepatic metastasis is curative and they present five-year survival in 70%; however, the use of somatostatin analogues is controversial. For metastatic and unresectable tumors, treatment will depend on the symptoms. If the patient is asymptomatic, somatostatin analogues will be used, although this is considered controversial when one suspects the progression of the disease. There are studies that show an improvement of symptoms with the use for short periods of time (less than three months). The symptomatic symptom control is mandatory [17,18,60].

Due the lack of early symptoms, patients with non-functional endocrine tumors of the pancreas (PNET) tend to present with much larger lesions than with functional tumors (4 cm vs. 1.9 cm) and, consult doctors in the late stages. Therefore, at the time of diagnosis, advanced metastatic disease is present [13,20,84,85,86]. For this reason, one study demonstrated that the five-year survival of malignant PNETs was lower in non-functional tumors (29%) than in functional tumors (41%) [87].

**Figure 9:**
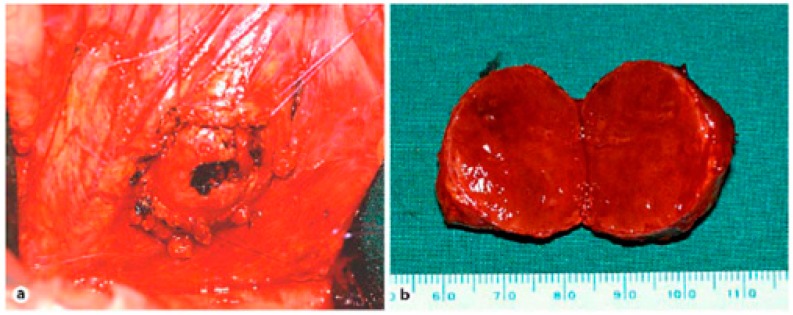
(**a**) Situsintra-operatory showing the tumor before enucleation. The tumor is flanked by four sutures that attach and enucleate from the surrounding tissue. (**b**) Specimen showing tumor 3 cm in diameter [26].

Unfortunately, curative surgical resection can only be performed in a minority of patients with pancreatic cancer (about 20%), since most have metastatic disease at diagnosis; in cases of PPoma, this percentage drops to less than 10% of eligible patients [10,13]. There are reports of cases of resection of PPoma tumors that present a well-defined solitary mass with benign histological characteristics resulting in good long-term survival (Figure 9) [25]. It is worth noting that total pancreatectomy, although resulting in a cure, is not indicated as it results in diabetes mellitus and exocrine pancreatic insufficiency. This procedure is only acceptable in patients from families with a high incidence of metastatic disease, because in this case, despite the consequences, total pancreatectomy can prevent early death [54]. There is also indication for cases of neuroendocrine tumors associated to MEN-1 [88,89]. It is important to emphasize that in patients with PPoma, they usually have distant metastasis, especially in the liver, meaning that they are considered incurable and therefore considered inoperable most of the time [12,13,17]. Another strategy is to submit patients to adjuvant chemotherapy (and sometimes radiation) treatment in order to reduce the risk of recurrence [14].

Recently, the technique of minimally invasive pancreatoduodenectomy (MIPS) as a laparoscopic pancreatoduodenectomy (LPD), has become a safe and viable alternative for benign tumors. However, this technique cannot be used for tumors in the head of the pancreas (where PPoma is located). The main concern regarding MIPS relies in blood loss, and although alternative, is a traumatic surgery proceeding [90].

With the identification of the *MEN-1* gene and mutations causing this syndrome, it was believed that it would be possible to identify the mutations associated with the malignancy of the pancreatic tumors and to identify patients at high risk of developing metastatic disease. However, mutational analysis of hundreds of families around the world did not demonstrate any significant phenotype–genotype correlation [91]. For the reasons mentioned above, the surgical goal is to remove the maximum tumor mass without a loss of pancreatic function [54]. In addition, tumor resection has shown an improvement or resolution of tumor-borne diabetes mellitus [52].

Since liver metastasis is common in PPoma, liver resection may be considered for localized disease without compromising organ function or extensive metastasis beyond the liver. Due to the indolent nature of most pancreatic neuroendocrine tumors, liver resection can lead to both long-term symptom relief and increased survival [12]. However, some authors consider treating patients with liver metastases to be somewhat controversial. In non-functional tumors less than 2 cm in diameter associated with MEN-1, surgery is not recommended [87].

In patients with predominantly hepatic disease, one strategy is embolization of the hepatic artery for those patients who are not candidates for surgery, observing response rates higher than 50%, although this is fleeting [12]. Thus, the combination of cytotoxic chemotherapy and local ischaemia, i.e., chemoembolisation, was evaluated in several studies. Symptomatic responses were obtained in most patients, whereas tumor shrinkage was observed in about half of patients with progressive disease prior to chemoembolisation [92]. Therefore, this procedure, which results in the ischaemia and necrosis of tumor tissue, has been useful for the reduction of hormonal hypersecretion syndromes [54]. The results of embolization show that symptom control and tumor growth occur in 90% of cases, leading to a five-year survival of up to 40%, and median survival of up to 32 weeks [12].

In cases where curative surgery is not possible, the debulking type should be considered. The advantages of cytoreduction (resection of up to 90% of the tumor volume must be achieved) include the reduced production of hormones by the tumor, control of symptoms and a decrease in tumor mass for the optimization of systemic chemotherapy doses. Clinical remission can be induced by palliative surgery, so the presence of lymph node and/or hepatic metastases is not necessarily a contraindication to surgery, obviously in the individual context of each patient. Thus, the cytoreduction of hepatic metastases with palliative intent to control symptoms increases median survival by 3–4 times, as well as providing excellent symptom control [12,93].

Radiofrequency ablation or cryoablation can be used for solitary liver lesions. Although less morbid, they can only be used in small lesions. Radiofrequency ablation in tumors <5 cm in size have shown 70–80% symptomatic responses with the control of symptoms for up to a year [17,85,94].

When surgical intervention is not indicated or when it does not result in the control of hormonal hypersecretion, patients are treated with drug therapy. This includes somatostatin analogues (octreotide and lanreotide), which have the power to inhibit the secretion of virtually all hormones, and from more specific treatments, which is not the case with PPoma [54]. Octreotide acts on somatostatin 2 and 5 receptors, inhibiting the release of neurohormones. It promotes optimal symptomatic control, with a significant improvement in the quality of life by up to 80%, a biochemical response of up to 70% and tumor stabilization in up to 20% of progression cases. However, the objective radiological decrease of the tumor occurs in less than 10% of cases. Therefore, the use of somatostatin analogues such as octreotide is highly effective in controlling signs and symptoms. Although octreotide may also decrease tumor size, evidence of its objective relationship is rare. Regarding the administration of octreotide, there is a need for reapplication every 8 h, which was supplanted by the availability of the deposit presentation, Sandostatin LAR^®^, which can be applied on a monthly basis. Long-term octreotide is initiated at a dose of 20 mg IM (intramuscular), with a gradual increase to the dose that is necessary for symptom relief after a period of sensitization. It is generally well tolerated; however, it has some side effects, such as nausea, abdominal discomfort, diarrhea and malabsorption of fats, which are mostly self-limited. In a randomized study, Sandostatin LAR^®^ at 10, 20 and 30 mg monthly doses was compared to daily octreotide SC (subcutaneous) (0.3–0.9 mg/day). The efficacy was similar in all groups in relation to control of the number of episodes of diarrhea (*p* > 0.72), and the 20 mg/month dose showed the best control of hot flushes. In patients who maintain symptoms at the dose of Sandostatin LAR 20 mg/month, dose escalation seems to rescue patients with initially resistant disease [12,17,95,96].

Systemic chemotherapy is used in patients with progressive metastatic disease. In this condition, patients with pancreatic islet tumors, where the PPoma is located, are more sensitive to systemic chemotherapy and therefore this is the treatment of choice. The chemotherapeutic agents used include streptozocin, doxorubicin, 5-fluorouracil (5-FU), dacarbazine and cyclophosphamide, but the responses are modest. The combination of streptozocin and doxorubicin is currently the chemotherapeutic regimen of choice; when compared to streptozocin alone, this combination improves the objective response from 36% to 65%, increases the mean duration of response to 20 months, and increases survival. Schemes combining doxorubicin and streptozotocin, with or without 5-FU, for four to six cycles, produce objective response rates ranging from 6% to 45%. Other agents such as taxanes and gemcitabine are relatively inactive in neuroendocrine tumors. Dacarbazine appears to be the single most active agent and is a good option for debilitated patients. In a phase II study of the Eastern Cooperative Oncology Group with 42 patients, an objective response rate of 33% was achieved. Like temozolamide, an alkylating agent was developed as a less toxic oral regimen than that of dacarbazine in a phase II study published in the Journal of Clinical Oncology in 2006, where twenty-nine patients were treated with a combination of temozolomide and showed a radiological response rate of 45%, which is comparable to that of studies of dacarbazine and streptozocin. It is important to emphasize that objective responses with chemotherapy can take up to four months to be documented [54,97,98].

Complete responses to chemotherapy treatment are rare and the vast majority of patients show recurrence. For this reason, and since it is generally an indolent process, chemotherapy should only be initiated when periodic monitoring demonstrates significant progression [54]. Against this background, new therapeutic approaches are being studied, such as the use of “target radiotherapy” and regimens incorporating the use of angiogenesis and tyrosine kinase inhibitors, such as sunitinib and gefitinib (Table 3). Sunitinib has been available in Brazil since 2006, and is used in the daily dose of 50 mg, VO (via oral), for four consecutive weeks every six weeks [12]. Therefore, when chemotherapy (sometimes along with a target therapy drug) prolongs survival for advanced disease, clinical trials are needed to further improve survival, and are currently in progress for several new agents [14].

In patients with octreotide (Octreotide-labelled Octreotide 111) hypercaption, especially when this uptake is greater than that of the hepatic parenchyma, the use of radioactive molecules coupled to a somatostatin analogue becomes a very attractive option. There are currently three radioactive compounds with different characteristics: 111-In-octreotide, 90-Y-octreotide and 177-Lu-octeotate. 111-In-octreotide has a lower tissue penetration, but in a small study with 16 patients (most with carcinoids) it showed a clinical benefit in 70% of the cases, for at least six months after the last application. In 30% of patients, the clinical benefit persisted for 18 months, without significant toxicities. 90-Y-octreotide has higher energy and, therefore, greater tissue penetration. Partial responses of the order of 8–29% were recently reported [12]. The most promising analogue appears to be 177-Lu-octreotide. Another study demonstrated that the octreotide somatostatin analogue has an affinity for somatostatin subtype 2 receptor that is nine times greater than octreotide. Using 177-Lu-octreotide in 131 patients with neuroenteropancreatic tumors resulted in a 2% partial response, with a median duration of response exceeding 36 months. In addition, a smaller response (reduction between 25% and 50%) was observed in more than 19% of the patients treated [99].

## 10. Conclusions

It is already fully accepted that the development of cancer involves the accumulation of genetic alterations. Knowledge and understanding of the genetic basis of cancer were mainly results of the rapid progress in molecular engineering.

Studies of the biology of pancreatic cancer have increased intensely making it very well-characterized. The demonstration that ductal adenocarcinoma presents distinct molecular characteristics compared to non-ductal and neuroendocrine tumors has important implications for the development of genetic theses, as well as for early diagnosis and prognostic markers.

Although there has been substantial progress in diagnostic imaging methods such as helical CT, endoscopic ultrasound associated with tumor markers and SPECT, there has been no significant improvement in cure rates in recent years.

It is hoped that with the prospect and availability of new technologies and the constant contributions of researchers in many related disciplines, we will obtain better results, not only for prevention and early diagnosis, but also to cure this fatal disease.

Against this background, PPoma has advanced from one of the most difficult objects of study, for which there was previously little genetic knowledge, to one of the best characterized human tumors. Therefore, the development of new diagnostic, preventive and treatment modalities requires an understanding of the molecular mechanisms of the complex multi-stage processes of tumorigenesis of the pancreas; research into these fields must therefore be performed.

## Figures and Tables

**Table 1:** Evolution in WHO terminology of neuroendocrine tumors of the digestive tract.

WHO 1980	WHO 2000	WHO 2010
**I. Carcinoid**	1. Well-differentiated endocrine tumor (WDET) *	1. Neuroendocrine tumor grade 1 (G1) (carcinoid)
	2. Well-differentiated endocrine carcinoma (WDEC) *	2. Neuroendocrine tumor grade 2 (G2)
	3. Poorly differentiated endocrine carcinoma/small cell carcinoma (PDEC)	3. Neuroendocrine carcinoma (large cell or small cell type)
**II. Mucocarcinoid**	4. Mixed exocrine-endocrine carcinoma (MEEC)	4. Mixed adenoneuroendocrine carcinoma (MANEC)
**III. Mixed forms carcinoid adenocarcinoma**		
**IV. Pseudotumor lesions**	5. Tumor-like lesions (TLL)	5. Hyperplastic and pre-neoplastic lesions

* The difference between WDET and WDEC was defined according to staging features in the WHO 2000 classification. G2 NET does not necessarily translate into WDEC of the WHO 2000 classification. Definition in parentheses for the International Classification of Diseases for Oncology (ICD-O) coding.

**Table 2:** Comparison of the criteria for the T category in the ENETS and UICC TNM classifications of pancreatic neuroendocrine tumors [81].

TNM	ENETS	AJCC/UICC
T1	Confined to pancreas, <2 cm	Confined to pancreas, <2 cm
T2	Confined to pancreas, 2–4 cm	Confined to pancreas, >2 cm
T3	Confined to pancreas, >4 cm, or invasion of duodenum or bile duct	Peripancreatic spread, but without major vascular invasion (superior celiac or mesenteric vessels)
T4	Invasion of adjacent organs or major vessels	Major vascular invasion (celiac axis or the superior mesenteric artery)

**Table 3:** Studies with new target therapies for neuroendocrine tumors.

Agent	Target	Patients	Response	PFS or TFP
Sunitinib	PDGFR; RET	66	17%	33 weeks (TFP)
Gefinitib	EGFR	31	6%	No response
Everolimus	mTOR	13	15%	No response
Temsirolimus	mTOR	15	7%	No response

PFS: Progression-free survival; TFP: Time for progression.
